# Exploring pathway interactions to detect molecular mechanisms of disease: 22q11.2 deletion syndrome

**DOI:** 10.1186/s13023-023-02953-6

**Published:** 2023-10-24

**Authors:** Woosub Shin, Martina Kutmon, Eleni Mina, Therese van Amelsvoort, Chris T Evelo, Friederike Ehrhart

**Affiliations:** 1 Department of Bioinformatics – BiGCaT, NUTRIM, Maastricht University, Maastricht, 6229 ER The Netherlands; 2 Maastricht Centre for Systems Biology (MaCSBio), Maastricht University, Maastricht, The Netherlands; 3 Leiden University, Leiden, The Netherlands; 4 Psychiatry & Neuropsychology, MHeNs, Maastricht University, Maastricht, The Netherlands

**Keywords:** Copy number variation syndromes, 22q11.2 deletion syndrome, Pathway analysis, Network analysis

## Abstract

**Background:**

22q11.2 Deletion Syndrome (22q11DS) is a genetic disorder characterized by the deletion of adjacent genes at a location specified as q11.2 of chromosome 22, resulting in an array of clinical phenotypes including autistic spectrum disorder, schizophrenia, congenital heart defects, and immune deficiency. Many characteristics of the disorder are known, such as the phenotypic variability of the disease and the biological processes associated with it; however, the exact and systemic molecular mechanisms between the deleted area and its resulting clinical phenotypic expression, for example that of neuropsychiatric diseases, are not yet fully understood.

**Results:**

Using previously published transcriptomics data (GEO:GSE59216), we constructed two datasets: one set compares 22q11DS patients experiencing neuropsychiatric diseases versus healthy controls, and the other set 22q11DS patients without neuropsychiatric diseases versus healthy controls. We modified and applied the pathway interaction method, originally proposed by Kelder et al. (2011), on a network created using the WikiPathways pathway repository and the STRING protein-protein interaction database. We identified genes and biological processes that were exclusively associated with the development of neuropsychiatric diseases among the 22q11DS patients. Compared with the 22q11DS patients without neuropsychiatric diseases, patients experiencing neuropsychiatric diseases showed significant overrepresentation of regulated genes involving the natural killer cell function and the PI3K/Akt signalling pathway, with affected genes being closely associated with downregulation of CRK like proto-oncogene adaptor protein. Both the pathway interaction and the pathway overrepresentation analysis observed the disruption of the same biological processes, even though the exact lists of genes collected by the two methods were different.

**Conclusions:**

Using the pathway interaction method, we were able to detect a molecular network that could possibly explain the development of neuropsychiatric diseases among the 22q11DS patients. This way, our method was able to complement the pathway overrepresentation analysis, by filling the knowledge gaps on how the affected pathways are linked to the original deletion on chromosome 22. We expect our pathway interaction method could be used for problems with similar contexts, where complex genetic mechanisms need to be identified to explain the resulting phenotypic plasticity.

**Supplementary Information:**

The online version contains supplementary material available at 10.1186/s13023-023-02953-6.

## Background

22q11.2 Deletion Syndrome (22q11DS), also known as DiGeorge or velocardiofacial syndrome (MIM:192,430), is a genetic disorder characterized by one copy of chromosome 22 missing a segment in the q-arm known as q11.2. In about 1 in 4000 live births an incident of 22q11DS [1, 2] is reported. Several somatic and neuropsychiatric symptoms are associated with this disease, including congenital heart defect, facial anomalies, schizophrenia/psychosis, hypoplastic thymus with immune deficiency, autistic spectrum disorder, palatal anomalies, neonatal hypocalcemia, speech and learning disabilities, and even combinations of such phenotypes [3–6]. The two most frequent phenotypes are congenital heart defect and schizophrenia, which comprises 60–70% and about 25%, respectively, of the patient population [7–9].

One major challenge of studying 22q11DS is to understand the molecular mechanisms between the deleted genes and the resulting disease phenotypes; previous research showed that the phenotypes vary widely among individuals, even though most patients share a common 3 Mb deletion [7, 10]. In addition, many of the associated clinical phenotypes are known to involve polygenic inheritance, for example autistic spectrum disorder, schizophrenia, and congenital heart defect, making it harder to track the propagation of this genetic perturbation. It is now suspected that multiple genes deleted within and outside the 22q11.2 region interact and target various cellular mechanisms, causing a range of clinical variation with different degrees of severity [11–14]. On the other hand, many studies on the mechanisms of 22q11DS up to this day focused on identifying associations between individual genes and certain phenotypes, and possibly as a result, some have reached inconclusive or inconsistent results. For example, the association between mutation of COMT in the 22q11.2 region and schizophrenia have been supported by some researchers but opposed by others [8, 75, 76]. Overall, much of the molecular dynamics of 22q11DS remain yet to be fully understood.

The complex genotype-phenotype variability of 22q11DS and the polygenic characteristics would benefit from novel bioinformatical approaches that utilize our current knowledge of complex interactions between biological materials. Previous efforts to organize this current knowledge by the bioinformatics community resulted in several large and publicly available databases, which can be easily exploited. In particular, pathway databases such as WikiPathways, Reactome, and KEGG, created from the literature and manually curated, delineate numerous biological interactions at the gene and molecular level [15–17]. An important feature of these repositories is that each pathway can be regarded as a module, a group of often co-regulated genes related to a common process [18]. Another important class of databases describes direct interactions between biological entities, such as those occurring between proteins (e.g. STRING) [19]. Using these information-rich databases, we can create a network that reflects our integrated understanding of how relevant genes and proteins regulate and interact with each other.

In addition, networks can help us integrate omics data and biological interactome data for further analysis [20]. Previous studies have demonstrated that network methods were effective in detecting several molecular-level features of cell functions that are associated with various diseases, such as cancer, cardiovascular diseases, neurological diseases, and many others [21–26]. For example, some researchers proposed examining direct interaction partners of known disease proteins, others checking topological characteristics such as hubs and modules [23, 27–29]. A notable number of algorithms apply path-based approaches, many of which use shortest paths or random walks, to identify disease genes and modules [28–30]. Previously, our group and others proposed a method to estimate interactions between pathways using threshold-based shortest paths traversing a biological network. This network was created from WikiPathways and KEGG repositories and was extended with protein-protein interaction and transcription factor-target information [31]. In that method, an interaction between two pathways is defined as a collection of significant paths, where two genes, each from different pathways, are connected via protein-protein or transcription factor-target interactions. The significant paths can then be collected and the calculated ‘pathway score’ is used to determine the strength of interaction between each pair of pathways [31].

The complexity of molecular features involving 22q11DS makes it an attractive subject for network biology methods discussed above. In addition, a deeper understanding of the genotype-phenotype relationships of 22q11DS may help us gain richer insights into several other diseases that display polygenic traits, such as neuropsychiatric diseases, by identifying the collective characteristics of interacting genetic variants [32–34]. In one of few studies in this subject, Jalbrzikowski et al. [37] analysed data of 22q11DS patients including those with autism spectrum disorder and/or psychosis, using weighted gene co-expression network analysis, and identified co-expressed gene modules associated with psychosis and autistic spectrum disorder. However, each module only provided the list of probes and information of associated genes, and more direct molecular relationships between the members, could have been established using our knowledge of biological interactions.

In this paper, we reimplemented Kelder et al.’s pathway interaction method to a transcriptomics 22q11DS dataset, originally published by Jalbrzikowski et al. [37], and demonstrated that, with proper adjustments, it could detect a gene subnetwork likely to be associated with phenotypic expression of autism spectrum disorder and/or psychosis among 22q11DS patients. Our approach used a combination of prior knowledge from WikiPathways, protein-protein interactions from STRING, and experimental data from a previous gene expression study regarding 22q11DS. We believe our approach can be flexibly applied to study molecular mechanisms of a wide range of genetic disorders with known origin - in our study, deleted genes at 22q11.2.

## Results

In this study, we re-analysed a transcriptomics dataset originally created by Jalbrzikowski et al. [37] using a novel pathway interaction analysis method to identify paths between the causative genes for this disorder and the differentially expressed genes.

### Candidate biomarkers

**Fig. 1:**
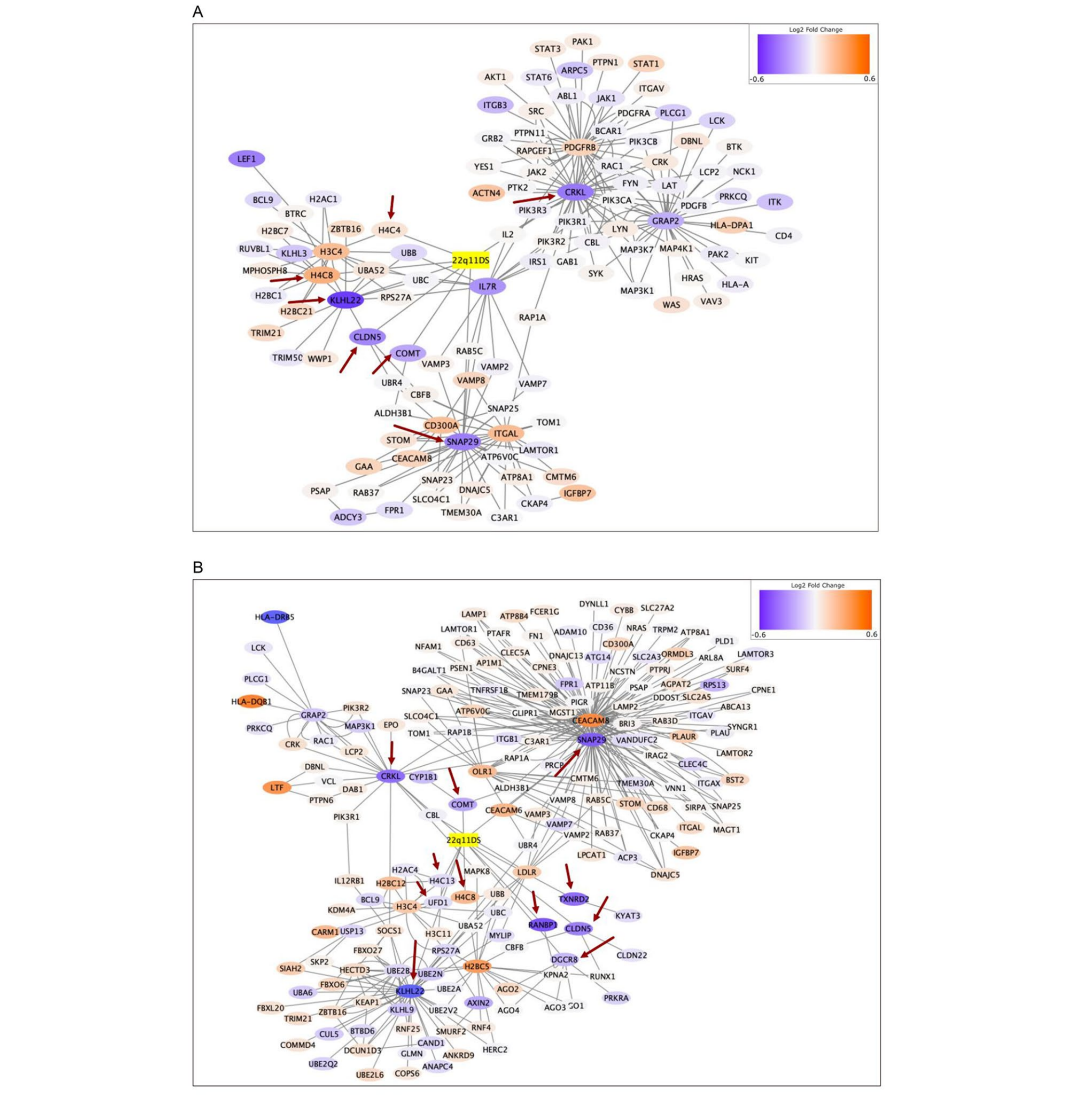
Pathway Interaction Network. Figure **1A** represents the significant path network of the Psychiatric Group, and Fig. **1B** the network of the Nonpsychiatric Group. Downregulated nodes were colored blue, upregulated red. The source node (yellow, labeled *22q11DS*) was colored yellow. Figure 1A shows several hubs such as CRKL, PDGFRB, GRAP2, and SNAP29, while Fig. 1B has CEACAM8, SNAP29, KLHL22, and OLR1 as most connected nodes. The genes that are known to be directly affected by the deletion, i.e., members of the 22q11DS pathway, are first neighbours of the source node and are marked with red arrows

After analysis of the transcriptomics dataset derived from peripheral blood, among a total 1,135 candidate pathways, the Psychiatric Group identified 167 pathways that included at least one significant path to the source (22q11DS pathway, WP4657), and 154 pathways with p-values less than 0.05. The Nonpsychiatric Group identified 246 significant pathways out of 255 pathways that have at least one significant path. The Psychiatric Group detected 116 genes contributing to the significant path network, and the Nonpsychiatric Group identified 185 such genes. The genes collected from the Psychiatric and Nonpsychiatric groups are presented in Fig. 1, with the source node, which was labeled *22q11DS* and colored yellow.

### Genes associated with phenotypic expression of neuropsychiatric diseases

**Fig. 2:**
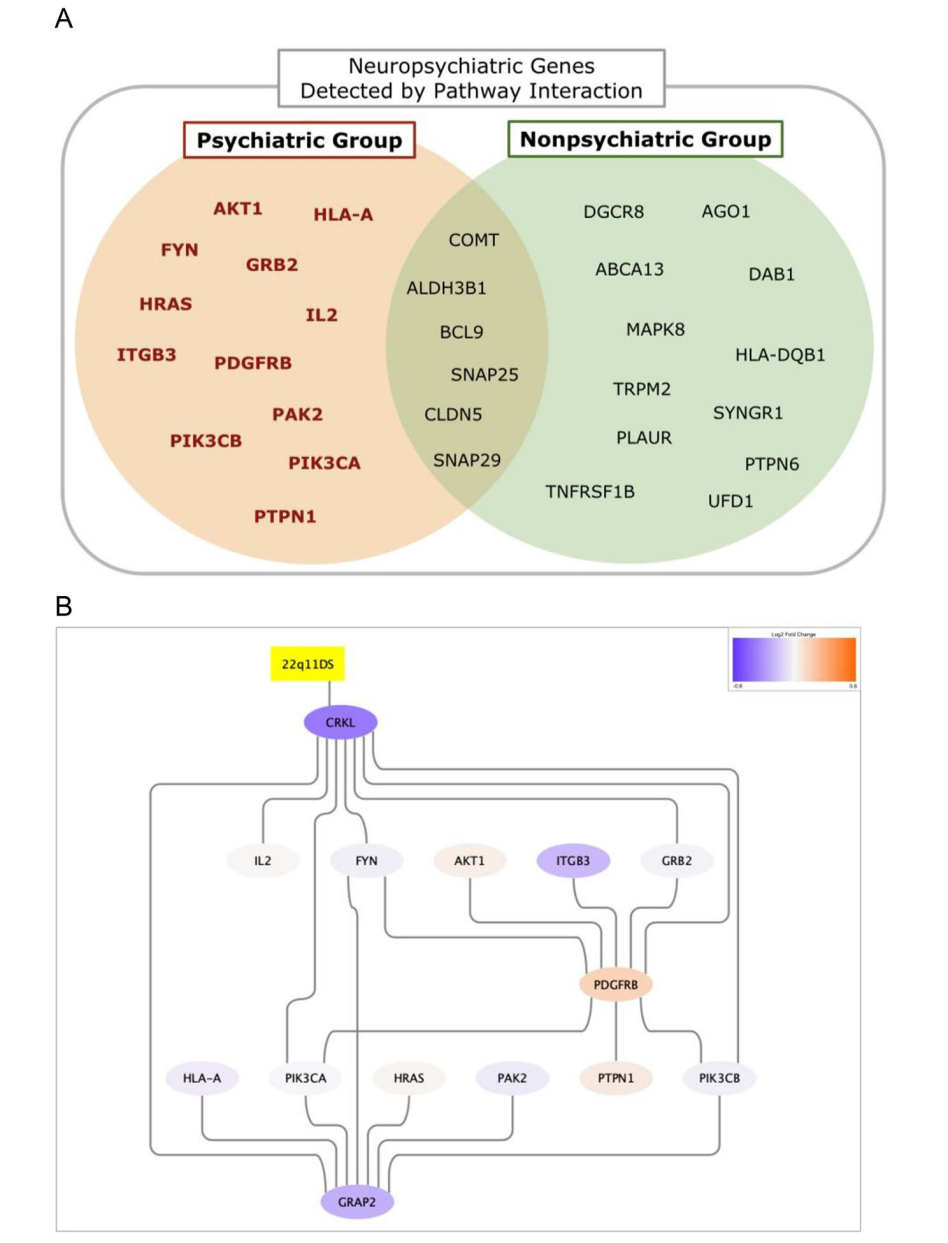
Neuropsychiatric genes detected by the pathway interaction network and their subnetwork. Figure 2A indicates the neuropsychiatric genes detected by two groups. The Psychiatric Group identified 12 exclusive neuropsychiatric genes, based on the classification in DisGeNet, included in the interaction network. Using this exclusive list of genes and two additional first-degree neighbors (CRKL and GRAP2), we were able to create a fully connected neuropsychiatric disease expression subnetwork shown in Fig. 2B. The 22q11DS pathway node was included to show its connection with CRKL. Supplementary Material S4 provides the full list of pathways that contain the detected genes

Our primary goal was to identify genes that were exclusively expressed among the autism spectrum disorder/psychosis patients. Figure 2 A describes the list of neuropsychiatric genes detected by the Psychiatric Group and the Nonpsychiatric Group. We found 12 neuropsychiatric genes that were exclusively represented among the patients experiencing autism spectrum disorder/psychosis. None of those genes were exactly located at 22q11.2, but their first-degree neighbors included CRKL, which is located within the commonly deleted region and is highly expressed in brain [11, 35]. Here, first-degree neighborhood means that such genes entail protein-protein interaction with CRKL. Using CRKL and an additional gene, GRAP2, one of the first-degree neighbors not in the DisGeNet list but suspected to be linked with schizophrenia [36], we were able to create a fully connected subnetwork with those 14 genes and visualized it as Fig. 2B. Supplementary Material S4 provides the full list of pathways that include any of the 14 genes.

### Pathways detected by pathway overrepresentation analysis (ORA)

Using the differential expression analysis for autism spectrum disorder and/or psychosis patients (Psychiatric Group), we found 217 differentially expressed genes, consisting of 98 downregulated and 119 upregulated genes. The same analysis involving patients without neuropsychiatric diseases (Nonpsychiatric Group) found 145 differentially expressed genes, among which the number of downregulated genes was 76 and that of upregulated genes was 69.

Table 1 lists top 10 pathways having at least two significant genes, detected from each data group. Pathway ORA from both Psychiatric and Nonpsychiatric data groups detected the 22q11DS pathway (WP4657, labeled as *22q11.2 copy number variation syndrome*) to be the most significantly overexpressed, which is rather obvious considering this pathway included the highest number of genes that had been directly affected by the deletion. Besides, only one pathway - *Transcriptional regulation of granulopoiesis* (WP5001) - was included in both lists.

**Table 1:** Top 10 pathways from pathway ORA from the Psychiatric Group (Table 1A) and that from the Nonpsychiatric Group (Table 1B). The pathways displayed here were ranked based on z score among those with at least three genes matching the criteria (p value < 0.05 and |log2FC| >= 0.15). Full lists are provided as Supplemental Material S5

wpid	pathway description	z score	p value
**A**			
WP4657	22q11.2 copy number variation syndrome	10.95	< 0.01
WP5001	Transcriptional regulation of granulopoiesis	6.81	< 0.01
WP2328	Allograft Rejection	6.30	< 0.01
WP4066	Interleukin-4 and Interleukin-13 signaling	6.26	< 0.01
WP3358	Caspase activation via Death Receptors in the presence of ligand	5.80	< 0.01
WP1829	Immunoregulatory interactions between a Lymphoid and a non-Lymphoid cell	5.17	< 0.01
WP3658	Wnt/beta-catenin Signaling Pathway in Leukemia	4.06	0.01
WP3350	TNFs bind their physiological receptors	3.85	< 0.01
WP69	T-Cell antigen Receptor (TCR) Signaling Pathway	3.84	< 0.01
WP4900	Purinergic signaling	3.59	< 0.01
**B**			
WP4657	22q11.2 copy number variation syndrome	14.71	< 0.01
WP5001	Transcriptional regulation of granulopoiesis	8.67	< 0.01
WP4101	Antimicrobial peptides	7.06	< 0.01
WP3391	Senescence-Associated Secretory Phenotype (SASP)	6.45	< 0.01
WP3359	DNA methylation	6.24	< 0.01
WP3364	SIRT1 negatively regulates rRNA expression	5.91	< 0.01
WP3397	Activated PKN1 stimulates transcription of AR (androgen receptor) regulated genes KLK2 and KLK3	5.80	< 0.01
WP3312	PRC2 methylates histones and DNA	5.52	< 0.01
WP3801	ERCC6 (CSB) and EHMT2 (G9a) positively regulate rRNA expression	5.34	< 0.01
WP4049	Neutrophil degranulation	5.24	< 0.01

Both data groups observed overrepresentation of biological pathways associated with immune system (e.g., *Allograft Rejection*-WP2328 and *Neutrophil Degranulation*-WP4049), general metabolism (e.g., *Purinergic signaling*-WP4900 and *DNA Methylation*-WP3359), and cancer-related pathways (e.g., *Wnt/beta-catenin Signaling Pathway in Leukemia*-WP3658), overall consistent with the findings by the authors of the dataset [37]. Pathways representing neuropsychiatric disorders, based on their titles, were not strongly represented in both results but the *Amyloid fiber formation*- *WP3547* pathway was found in both groups. The complete results and associated genes were provided as Supplementary Material S5.

**Fig. 3:**
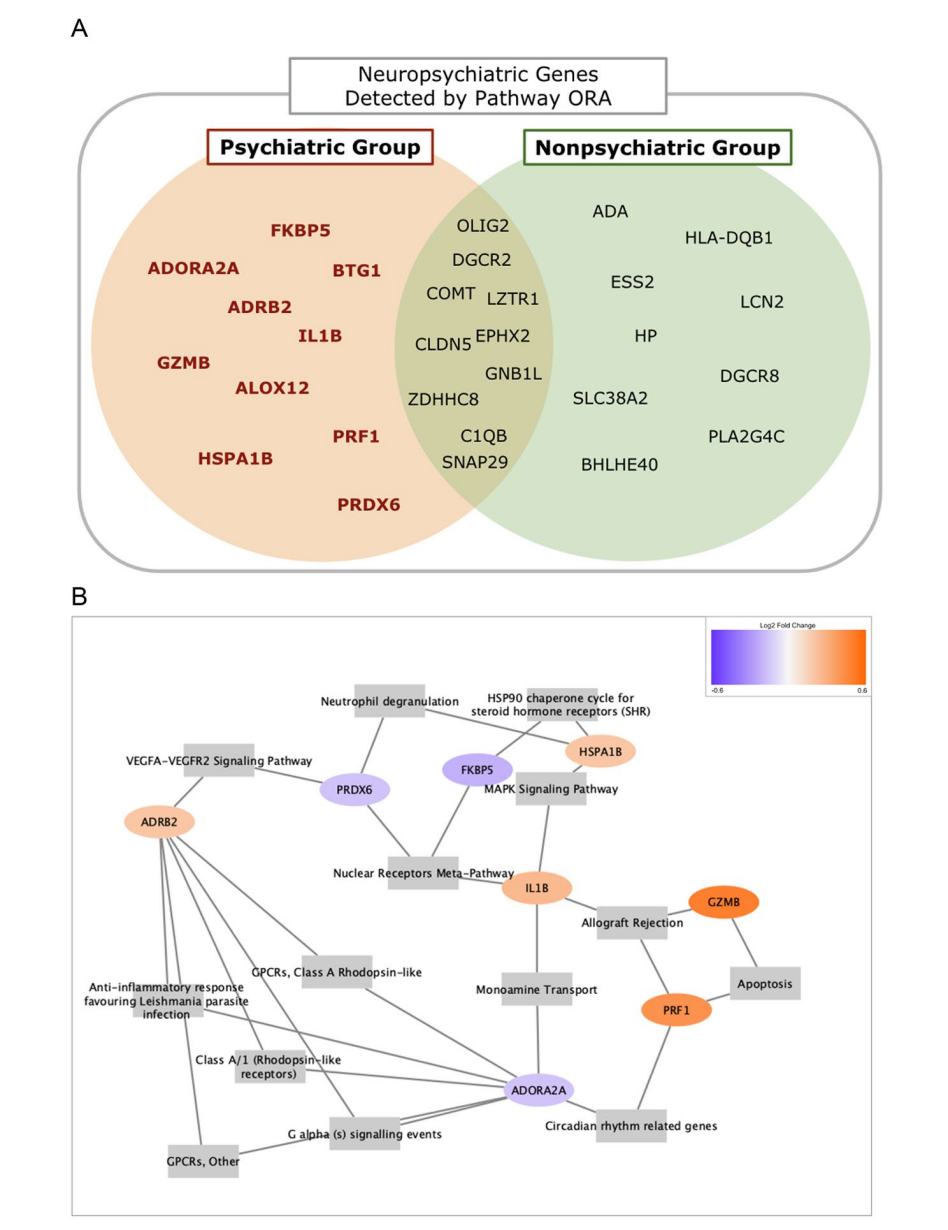
Neuropsychiatric genes detected by Pathway ORA. The Psychiatric Group identified 10 exclusive neuropsychiatric genes that were differentially expressed, as shown in Fig. 3A. Figure 3B is a gene-pathway network of the pathways that contain two or more such genes. ALOX12 and BTG1 were not included in any such pathways

The overrepresentation analysis using differentially expressed neuropsychiatric genes is shown in Fig. 3. The genes identified by the two data groups were represented as a Venn diagram in Fig. 3A. Compared to the pathway interaction method, pathway ORA identified a different list of exclusively differentially expressed neuropsychiatric disease genes. Three genes - COMT, CLDN5, and SNAP29 - were commonly identified by both the pathway interaction and the pathway ORA methods, and HLA-DQB1 and DGCR8 were exclusively identified among the Nonpsychiatric Group by both methods. All these genes, except CLDN5, are also expressed in brain tissues as compared with data from The Human Protein Atlas [https://www.proteinatlas.org/]. Figure 3B visualizes the exclusively differentially expressed genes by the Psychiatric group as a gene-pathway network, using the pathways that include two or more such genes. Most pathways were connected to ADRB2 and ADORA2A, genes mainly associated with G-protein signalling pathways and expressed in the brain. FKBP5, GZMB, and PRF1 showed the highest expression level (log2FC), however, GZMB and PRF1 are typically expressed in blood, not brain tissue. The full list of pathways including these genes is provided as Supplementary Material S6.

## Discussion

The pathway interaction method, originally proposed by Kelder et al. [31], analyses biological networks, constructed by previously identified relationships between genes and examines their collective behaviors. In this paper, we demonstrated that this method could detect the disease expression subnetwork (in this study, clinical expression of autism spectrum disorder and/or psychosis) that was likely to have been affected by the deletion in the 22q11.2 region. The summary of our workflow is as follows. First, we created two large directed graphs, one for patients with autism spectrum disorder/psychosis and the other without, consisting of protein-protein interactions and the WikiPathways repository. Next, using two transcriptomics datasets of 22q11DS, we identified sets of pathways and genes that closely ‘interact’ with the 22q11DS pathway (*22q11.2 copy number variation syndrome*, WP4657). We then refined the interaction networks and identified a gene subnetwork that was exclusively associated with 22q11DS patients experiencing autism spectrum disorder/psychosis. The results were compared with that of pathway ORA.

In the subnetwork shown in Fig. 2B, a notable relationship is a propagation of a signal starting from CRKL, a commonly deleted gene in the 22q11.2 region, leading to GRB2, and then PDGFRB, which is a major hub within the subnetwork. Previous studies suggest that CKRL, GRB2, IL2, ITGB3, and FYN are involved with natural killer (NK) cell activation [38–42]. Indeed, previous research observed decreased NK cell function among 22q11DS patients [43]. In addition, there have been several studies supporting that reduced NK cell cytotoxicity is associated with neurodevelopmental disorders, especially autism spectrum disorder [44–46]. Consequently, we suspect that NK cell function might be one of the important biological processes disrupted among the 22q11DS patients who experienced neuropsychiatric disorders. The upregulation of PDGFRB might give another piece of evidence for this mechanism. PDGFR-DD binds to NKp44 to activate NK cells, and also interacts with PDGFRB, to stimulate further downstream pathways [47–49]. Therefore, the upregulation of PDGFRB might be the result of increased PDGFR-DD activity, via a possible feedback loop, to compensate for the reduced NK cell functions initially caused by the silencing of CRKL.

Besides the association with NK cell functions via PDGFRB, a number of studies reported that the downregulation of the PI3K/Akt pathway is associated with autism spectrum disorder and schizophrenia [50–53]. PIK3CA and PIK3CB, two class I subunits of PI3K, as well as AKT1 are thus likely to have influenced the phenotypic expression of autism spectrum disorder and/or psychosis among the 22q11DS patients. Since GRB2 plays an important role in PI3K activation pathways [54, 55], GRAP2 (GRB2 related adaptor protein 2) can be considered to take part in the same pathway. There have been a few studies which suggested the association between GRB2/GRAP2 and schizophrenia [56–58], but we suspect GRAP2 might be actively involved in integrating and transmitting the effects of gene deletion leading to the expression of neuropsychiatric diseases. The role of other genes, HLA-A, HRAS, and PAK2, whose connection was not clearly demonstrated by the disease subnetwork, may be illuminated in conjunction with GRAP2 or its close interaction partners.

We can summarize our findings as follows. The differentiating feature of the Psychiatric Group over the Nonpsychiatric Group, i.e. the molecular signature of the 22q11DS patients who have experienced autism spectrum disorder and/or psychosis, compared to those who have not experienced such diseases, implies the involvement of NK cell functions and PI3K/Akt signalling pathway, as well as several genes such as CRKL, PDGFRB, and AKT1.

**Fig. 4:**
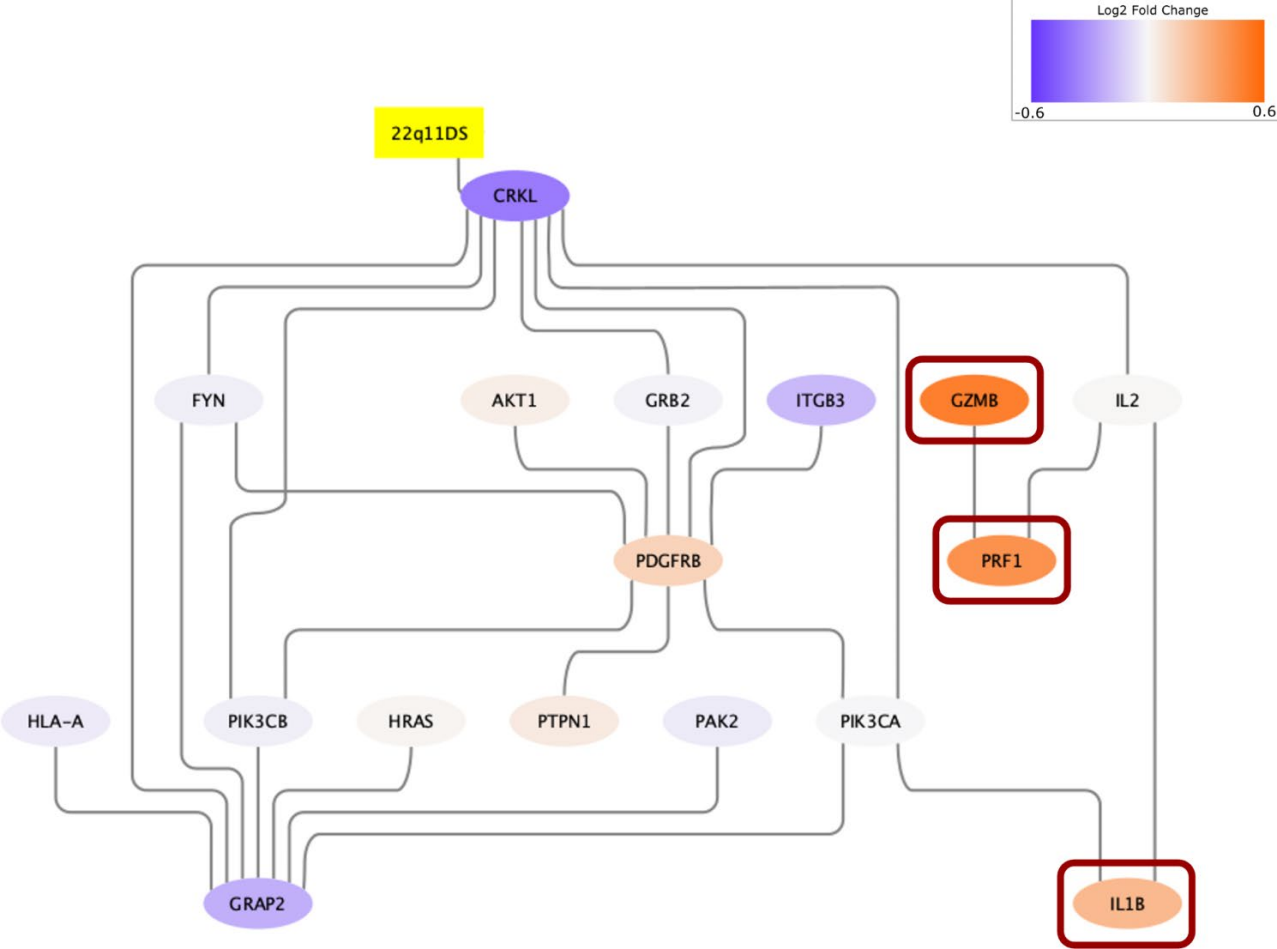
The extended molecular interaction network. The figure represents the combined subnetwork from the pathway interaction analysis and pathway ORA, of the genes suspected to be exclusively affected among 22q11DS patients experiencing neuropsychiatric diseases. All edges shown here, except 22q11DS-CRKL, indicate protein-protein interactions. GZMB, PRF1, and IL1B (in red box) were identified by pathway ORA and had direct protein-protein interactions with some of the nodes identified from the pathway interaction subnetwork. The other ORA genes did not have any connection with the current subnetwork according to our STRING information

Compared to the pathway interaction method demonstrated here, pathway ORA detects individual genes that are significantly differentially expressed and are contained in previously defined pathways. Among the identified significantly expressed neuropsychiatric genes, current literature suggests that PRF1, GZMB, ADORA2A, ADRB2, and IL1B play important roles regarding the NK cell function [59–63]. On the other hand, PRDX6, HSPA1B, and FKBP5 are known as biomarkers involved in regulation of the PI3K/Akt signalling pathway [64–66]. Therefore, we can conclude, at the level of biological processes both the pathway interaction and the ORA methods imply certain biological pathways that are commonly involved, which may warrant closer look at such mechanisms in future study. Considering the most significantly differentially expressed genes were a bit far from the deleted genes of the 22q11.2 region, we suspect some negative feedback mechanisms that decrease the expression level of the genes that directly interact with both the deleted genes and the most significant genes. Still, we found that three of the genes (GZMB, PRF1, IL1B) from pathway ORA had direct protein-protein connections with the genes in Fig. 2B. Using this, we further extended our network, which is displayed in Fig. 4.

It would be beneficial to briefly compare the two methods we used. Considering our initial goal was to identify molecular interactions associated with the expression of autism spectrum disorder/psychosis among the 22q11DS patients, the pathway method seemed to offer a convincing way to identify a molecular subnetwork of the disease. One significant advantage of our method over the conventional pathway ORA is that the pathway interaction method can collectively detect genes of interest, where the connections between such gene sets indicate plausible biological relationships powered by prior knowledge instead of “guilt by association”. For example, GRB2, IL2, PIK3CA, and PIK3CB were not differentially expressed enough under the individual gene significance criteria, thus would not have been identified had we applied only pathway ORA, even though they have direct protein-protein interactions with other significant genes. On the other hand, pathway ORA seems to be better at identifying individual genes that are strongly expressed, regardless of the expression level of nearby genes. Another advantage of ORA is that gene significance criteria can be easily adjusted and modified, and can easily include any combination of variables, while the pathway interaction method depends only on gene weights, which first needs appropriate transformations. Our understanding is that the two methods complement each other and together reveal a more complete picture of the molecular interaction of 22q11DS, as shown in Fig. 4. Therefore, future research projects would benefit from exploiting both methods. Table 2 summarizes the characteristics of the two methods.

**Table 2:** Comparison of the pathway interaction method and pathway ORA. Here we summarized a number of differences and similarities between the pathway interaction method and pathway ORA. As can be seen below, they differ in the databases used, number of genes detected, the way significant genes are selected, and the structure of resulting subnetworks. On the other hand, in our study both methods identified the same biological processes. The two have different strengths and weaknesses; we suggest that researchers exploit both methods to gain a bigger picture of the biological mechanism

	Pathway Interaction	Pathway ORA
Database Used	WikiPathways, STRING	WikiPathways
Detected Unit	Significant path (two or more genes connected by protein-protein interaction)	Individual gene
Selection Criteria	Sum of weights (transformed T-statistics)	P-value and log2FC score
Resulting Subnetwork	Pathway-gene-gene-pathway	Gene-pathway-gene-pathway
Biological Processes Identified for Psychiatric 22q11DS	NK cell function PI3K/Akt signalling	NK cell function PI3K/Akt signalling
Strength	Can detect multiple genes collectively having a strong expression profile, backed by prior knowledge.	Can detect single strongly expressed genes using flexible criteria.
Weakness	Strongly dependent on pre-existing relationships between gene products. Genes with unidentified relationships will be ignored. Detection criteria are hard to adjust or modify.	Individual gene selection can be highly dependent on the significance criteria. Depending on data quality, spurious genes might be mistaken as being significant.

For our study, we have applied several adjustments to the original methods by Kelder et al. [31]. First, the “interaction” between all possible pairs of two pathways was redefined as a source-target relationship, where the source was fixed as the 22q11DS pathway, and the focus was to choose the appropriate target pathways that interact with the source, rather than describing interaction signatures between all pairs. Next, the path threshold was initially set at 0.9 when selecting significant pathways, but then flexible threshold criteria were applied to collect actual paths, backed by our sensitivity analysis on threshold-score relationships. Flexible criteria were used to detect additional pathways that involve genes that did not have a large change, but still contributed to the paths by interacting with other significant genes. Calculating empirical p-values was also simplified; 1,000 samples rather than 10,000 samples, and each sample matches the number of genes having weight less than or equal to 0.8 and those having weight greater than 0.8. In addition, our focus was to offer a plausible interpretation of molecular mechanisms starting from an actually deleted gene on 22q11.2 (in our dataset, CRKL), by comparing results obtained from two parallel analyses of the Psychiatric and the Nonpsychiatric Groups. As for the final step, we compared our results with that of pathway ORA and demonstrated that the two methods could complement each other to obtain a more complete picture of the molecular perturbation from the gene deletion.

The value of pathways in this context is that pathways offer relatively safe start and end points. Pathways (at least those in the WikiPathways repository) are created and supported by previous publications, so that analysis revolves around plausibly grouped genes, making the results easy to interpret. Computationally speaking, pathway nodes are excellent source and target nodes, since it is much more efficient to find significant paths between pathways than to examine paths between all candidate gene pairs. In our study, we were able to use an already existing pathway (22q11DS pathway) as the source node, as it incorporated the deleted genes and their close interaction partners that were previously known. However, we would like to emphasize the flexibility of our method as well, since even if no published pathway existed, one would be able to directly create an appropriate gene-pathway relationship and use it as a starting point. The pathway interaction method can also include transcription-factor targets or other types of interaction databases, in addition to or instead of protein-protein interactions to further extend the base network.

As a final note, we would like to briefly discuss the data used. The dataset was obtained from whole blood rather than from brain cells, implying the tissue-specific gene expressions might have affected the key genes and pathways identified; for example, we found pathways involving innate immune systems and cell metabolism to be significantly over-expressed in both data groups. Considering our goal was to study the phenotypic expression of autism spectrum disorder and psychosis, actual brain cell expression data may provide clearer pictures on the dynamics between the deleted genes and related biological processes, for example, whether the perturbations in the cellular processes we observed was due to peripheral pathology or a secondary effect, as result of brain dysfunction. However, our dataset was still useful to examine the neuropsychiatric genes that might reveal crucial interactions via the immune-related responses. The research community also has discovered that neuropsychiatric functions and immune-related pathways tend to be closely associated [67, 68]. We hope future experimental work would confirm and verify our findings.

## Conclusions

Challenges to understanding exact mechanisms of 22q11.2 Deletion Syndrome (22q11DS) originate from the high level of genotype-phenotype variability and the polygenic inheritance of the disease. In this paper, we explored the pathway interaction method to identify molecular signature of 22q11DS, especially that of patients experiencing autism spectrum disorder and/or psychosis. Using the method, we were able to identify and explain possible involvement of pathways including the NK cell functions pathway and genes such as CRKL for the development of autism and/or psychosis in 22q11DS patients. Our approach is flexible and can incorporate various knowledge of molecular dynamics, such as protein-protein interactions, and can be used to study other rare diseases with a similar context, where complex genetic mechanisms between mutated genes and the resulting phenotypes are in question.

## Methods

### Data preparation

For this study we used a publicly available transcriptomics dataset of whole-genome Illumina Human HT-12 microarray assays, obtained from whole blood RNA of a total 112 subjects (46 patients and 66 healthy controls), from a study originally published by Jalbrzikowski et al. [37], with GEO accession number GSE59216. In the dataset, 46 individuals were 22q11DS patients, among which 19 had a diagnosis of either autism spectrum disorder (N = 16) or psychosis (N = 6). Three patients were diagnosed with both. In this dataset, the proportion of patients with autism spectrum disorder and/or psychosis was significantly higher (41.3%) than that of the general patient population, which is about 25% [7]. The preprocessed dataset was downloaded from GEO. In order to detect genes and their interactions distinctive among patients with autism spectrum disorder/psychosis, we created two datasets from the original data. The first set included data of healthy controls vs. 22q11DS patients *with* autism spectrum disorder/psychosis (N = 66 + 19 = 85), and the second set contained that of healthy controls vs. patients *without* reported psychiatric symptoms (N = 27 + 66 = 93). We analysed the two datasets separately, using the approaches described below, and compared the results.

We decided to conduct two separate analyses instead of directly comparing the patients *with* vs. *without* neuropsychiatric phenotypes. First, we noted that the number of patients with neuropsychiatric phenotypes [19] and the number of patients without such phenotypes [27] were rather small, so direct comparison of the two groups may not yield meaningful coefficients and p-values due to the small sample size. Second, both phenotype groups have gene deletion at similar locations but showing different phenotypes; we reasoned that it might be useful to identify genes that are commonly expressed in both groups and even the genes identified only by the non-neuropsychiatric group, especially for future studies. Combining healthy controls with each group for analysis and comparing their results would help address these points.

We used R version 4.0.3 for our data analysis. As an initial step, we conducted a differential expression analysis at probe-level using the limma R package, version 3.46.0 [69]. Gene-level log2 fold change (log2FC), p-values, and T statistics were collected by taking the probe observation with the median log2FC score, out of the probes matching each NCBI Entrez ID. In addition, a new variable, called *weight*, was introduced from T statistics using the formula below, as suggested by Kelder et al. [31]. The weight indicates the edge weight of the pathway network, constructed in the next section.

$$weight = 1-\frac{1}{1+{e}^{-2\left(\right|T|-3)}}$$

The weight variable inversely maps T statistics, which can take any real number (-infinity, +infinity), into a value within (0, 1). A smaller value of weight indicates a large T-value, which can be interpreted as a possibly significant level of gene expression, and vice versa. According to Kelder et al., genes with an absolute T statistic > = 3 correspond to unadjusted p-value < = 0.004 [31].

### Analysing interactions between pathways

**Fig. 5:**
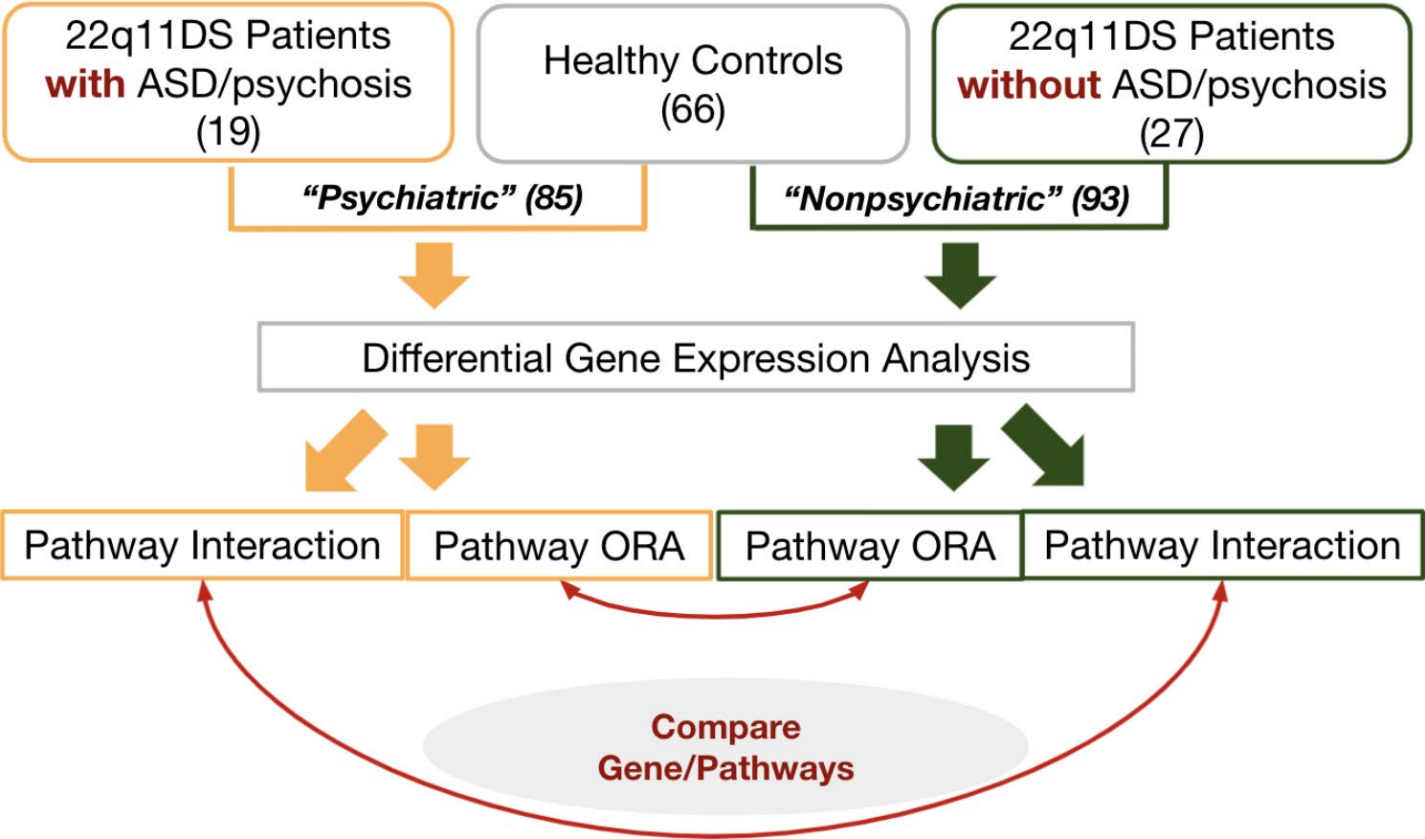
A summary of workflow. First, we created two subgroup datasets (Psychiatric vs. Nonpsychiatric) and conducted a differential expression analysis for each set. Next, the pathway interaction method and pathway over representation analysis (ORA) were applied to each set of differential expression analysis results. Relevant genes and biological processes associated with neuropsychiatric diseases were identified by comparing the results from the pathway interaction method and pathway over representation analysis, respectively, by creating relevant networks. Finally, subnetworks from the two methods were compared and discussed. (ASD = autism spectrum disorder, 22q11DS = 22q11.2 deletion syndrome)

The pathway interaction analysis mostly followed the proposed method by Kelder et al., but we have made several adjustments in later steps to improve biological interpretability and contextual applicability. The original method by Kelder et al. was described visually as Supplementary Material S1, directly from the study ([31] Fig. 5). We will discuss the novel aspects of our new approach in the discussion section in detail. The first step was to construct a network on which the paths would be identified. We started with creating a gene-pathway network using 628 pathways from the WikiPathways repository (Version 20,210,110) and 508 WikiPathways-archived Reactome pathways (Version 75). From the pathways, all genes with expression data were included. This network was extended by protein-protein interaction pairs that have a combined score of 900 or above from the STRING database Version 11.0 [19]. Between each pair of nodes (gene-gene or gene-pathway pair), two directed edges, each pointing at the opposite side, were added, with the edge weight being the weight of the edge head, calculated earlier using the T statistics and the formula above. If the edge was heading at a pathway node, zero was assigned to its corresponding edge weight.

In the network constructed above, we called the 22q11DS pathway node (labeled WP4657) the *source*, and identified ‘significant paths’ that connected the source with another pathway node. A significant path was defined as a directed path comprising three or more edges, starting at WP4657 and ending at another candidate pathway node, with the edge weight sum below a certain threshold (l_max_); the abbreviation ‘l’ originated from weighted ‘length’, which indicates the sum of edge weights). The minimum number of edges was introduced in order for each connected pair of pathways to include at least one protein-protein interaction. We applied the following algorithm to each candidate pathway (The difference in the below algorithm between the original method is that we only considered the pairs including the source, rather than all possible pathway pairs.):

1. Find the shortest path between the 22q11DS WP4657 pathway and the candidate pathway.
2. Calculate the sum of edge weights of the shortest path, identified in step 1 above; if the sum is greater than l_max_, stop the process; pick the next candidate pathway, and return to [1].
3. If the sum is less than l_max_, check the length (number of edges) of the path, i.e. the number of edges.
  - If length is 2, remove the last edge.
  - If length is greater than 2, remove all edges except the first and the last edge.
4. Store the path information, pick the next candidate pathway, and return to [1].

Using the algorithm above, we first collected all pathways that had at least one significant path connected to the central 22q11DS pathway (the “source”). The list of pathways depends on the path threshold l_max_; we first used 0.9 which was suggested by Kelder et al., i.e., a pathway would be selected if it contained at least one path with the sum of edge weights less than 0.9. Next, each candidate pathway was assigned an interaction score, using the formula below, where n was the total number of significant paths and l_i_ the weight sum of each significant path.

$$Scor{e}_{}={\sum}_{i=1}^{n}\frac{1}{{l}_{i}}$$

For pathways with at least one significant path, we also calculated empirical p-values to check the statistical significance, since pathways with large numbers of genes were likely to have many significant paths, resulting in high crude interaction scores. For each selected pathway, we created 1,000 modified networks, where the corresponding gene-pathway portion was replaced by the same number of randomly chosen genes. In each sample, the proportion of differentially expressed genes with weight below 0.8 was fixed, so that they are likely to contribute to significant paths if there exist appropriate protein-protein interactions. After calculating 1,000 random interaction scores from the networks, we computed the p-value of the pathways using the number of incidents that yielded the interaction score greater than or equal to the original score [70]. Those having p-values less than 0.05 were selected for further consideration. The list of pathways and corresponding p-values were included as Supplementary Material S2.

Once the list of pathways was finalized, we collected significant paths, this time with a relaxed threshold. The reasoning behind this was that we wanted to identify paths containing a relatively non-significant gene that still interacts with other genes that were significantly expressed. Expression of a gene is not constant over time and can be affected by activities of various other genes that interact with it, therefore, we wanted to find such genes (hence a meaningful collection of genes that interact with each other) whose partners had been significantly activated or repressed. Finally, all collected paths were filtered using the final threshold: l_max_=0.9 for three-edge paths, and l_max_=1.4 for longer ones. The reason was that, when the threshold l_max_ increases, we want to remove shorter paths with large weights. The detailed reasoning of the relaxed threshold was based on our sensitivity analysis, which is included as the Supplementary Material S3.

In the final result, each selected pathway contained a pathway score and a list of genes that were connected via its significant paths. The resulting subnetwork was visualized using Cytoscape version 3.8.1 [71]. Neuropsychiatric genes from the pathways that closely interacted with the source node were identified and examined with existing literature.

### Detection of key pathways and genes associated with autism spectrum disorder/psychosis

A major goal of our analysis was to identify meaningful biological interactions and key genes associated with autism spectrum disorder and psychosis of 22q11DS patients. To achieve this, we created two datasets from the GSE59216 dataset and ran two separate analyses using the approach described above. The first dataset included 22q11DS patients *with* autism spectrum disorder/psychosis and healthy individuals (Psychiatric 22q11DS vs. Healthy Control Group, we call it *Psychiatric Group*); the second group contained 22q11DS patients *without* autism spectrum disorder/psychosis and healthy individuals (Non-psychiatric 22q11DS vs. Healthy Control Group, we call it *Nonpsychiatric Group*).

We identified the characteristics of genes and pathways derived from the Psychiatric Group, which were not detected by the Nonpsychiatric Group. To ensure certain genes were indeed associated with neuropsychiatric diseases, we downloaded the ‘Curated gene-disease associations’ dataset from the webpage of DisGeNet Version 7.0, published in January 2020 [72], and extracted the list of genes labeled as ‘schizophrenia’ (UMLS CUI: C0036341), ‘autistic disorder’ (UMLS CUI: C0004352), and ‘psychotic disorders’ (UMLS CUI: C0033975). Using this list, we selected the genes identified by the significant path networks from the two data groups and compared the results. This would illustrate the 22q11DS-related biological processes that led to the phenotypic expression of autism spectrum disorder/psychosis.

### Comparison with pathway overrepresentation analysis

In the final step of our study, we compared the results of the pathway interaction method with that from the pathway overrepresentation analysis (ORA). Pathway ORA is an enrichment method which identifies pathways containing significant numbers (i.e. higher numbers than expected) of genes of interest, assuming such genes follow hypergeometric distribution [73]. First, each gene was regarded as significantly differentially expressed if (1) the p-value of it was less than 0.05 and (2) the absolute value of log2FC was greater than or equal to 0.15. These criteria were chosen in order for the number of significant genes to roughly match the number of genes detected by pathway interaction networks, as well as to yield relatively easy interpretation (for example, 2^0.15^ would indicate about 10% up-regulation compared to healthy controls). Next, with those differentially expressed genes, we used PathVisio to conduct pathway ORA [73, 74]. In addition, we again used the same DisGeNet dataset to identify differentially expressed genes that were also associated with neuropsychiatric diseases. Such genes and the pathways that contained them were visualized as a network.

We note in passing that when conducting the overrepresentation analysis and selecting pathways from the interaction network, we used p-values instead of adjusted p-values. First, the reason was to make the analysis overall consistent with the original method proposed by Kelder et al., where the weights were computed using T-statistics, a direct basis of calculating p-values without further scaling. Second, in our overrepresentation analysis, p-value and log2FC score thresholds were selected so that the number of detected genes roughly match that from pathway interaction analysis, rather than to determine the statistical significance of the genes. In our future studies, we are interested in extending our analysis to give the full scope of weighting schemes as well as gene- or pathway-selecting criteria.

An overview of our approach is summarized in Fig. 5. The scripts we used for analysis can be found at https://github.com/woosubs/PathwayInteraction.

### Electronic supplementary material

Below is the link to the electronic supplementary material.

Supplementary Material 1

Supplementary Material 2

Supplementary Material 3

Supplementary Material 4

Supplementary Material 5

## Data Availability

The data was taken from a previously published study by Jalbrzikowski et al., with GEO accession number GSE59216. The analysis scripts can be found under https://github.com/woosubs/PathwayInteraction.
