# Supplementary material for: Exploring pathway interactions to detect molecular mechanisms of disease: 22q11.2 deletion syndrome
Source: Orphanet J Rare Dis. 2023 Oct 24;18:335. doi: 10.1186/s13023-023-02953-6 (PMC10594698; doi:10.1186/s13023-023-02953-6)
Supplement: Supplementary file 1 — Supplementary Material 1 [file 13023_2023_2953_MOESM1_ESM.docx]

The figure and explanation below were directly from Figure 1 of **Kelder, Thomas, et al. “Exploring Pathway Interactions in Insulin Resistant Mouse Liver.” BMC Systems Biology, vol. 5, Aug. 2011.**


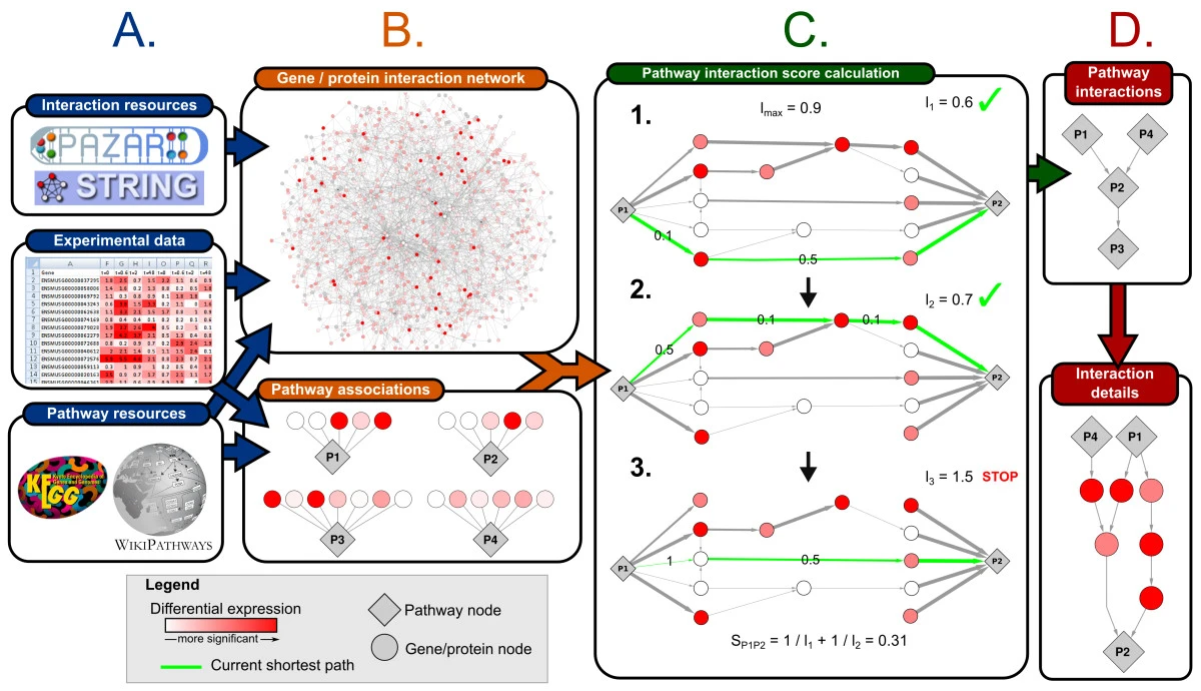


**Overview of the analysis approach to investigate interactions between pathways**. A: Information from different resources and experimental data is integrated into a weighted gene/protein interaction network and a set of pathways and their associated genes and proteins. B: Based on the interaction network, an interaction score and significance is calculated for each pathway pair. C: Example of the process of identifying a set of non-redundant shortest paths for the interaction of pathway P1 to P2. This panel shows step 5-7 of the calculation as described in the Methods section. D: Two representations of the resulting pathway interactions. The top panel shows the pathway interaction network, where each edge represents a significant interaction between two pathways. The bottom panel shows a detailed network showing the identified shortest paths between pathways P1, P4 and P2.
