# Supplementary material for: Exploring pathway interactions to detect molecular mechanisms of disease: 22q11.2 deletion syndrome
Source: Orphanet J Rare Dis. 2023 Oct 24;18:335. doi: 10.1186/s13023-023-02953-6 (PMC10594698; doi:10.1186/s13023-023-02953-6)

Supplemental Material S2: We increased the maximum path threshold l_max_, starting from 0.9 to 1.8, by 0.1, and calculated the total sum of interaction scores of all candidate pathways. The total interaction score of the Non-psychiatric group jumped at l_max_=1.0, then increased marginally until l_max_=1.4, after which it started to grow quickly. The score of the Psychiatric group increased at l_max_=1.0, then slowly went up afterwards. Since we wanted to include additional paths with not too many spurious ones, We relaxed the threshold to l_max_=1.4 for paths with four or more edges for both groups.

A: Total pathway score increases for Psychiatric Group and Non-psychiatric Group.

| Threshold | Psychiatric Group | Non-psychiatric Group |
| --- | --- | --- |
| 0.9 | 383.22 | 641.89 |
| 1.0 | 2248.01 | 2831.82 |
| 1.1 | 2657.44 | 3209.79 |
| 1.2 | 2716.60 | 3602.78 |
| 1.3 | 2883.62 | 3926.93 |
| 1.4 | 3828.23 | 5166.27 |
| 1.5 | 4033.44 | 6716.53 |
| 1.6 | 4487.30 | 10272.08 |
| 1.7 | 4939.80 | 15166.71 |
| 1.8 | 6197.95 | 21645.99 |

B: Dot plots describing the increase of total pathway scores.


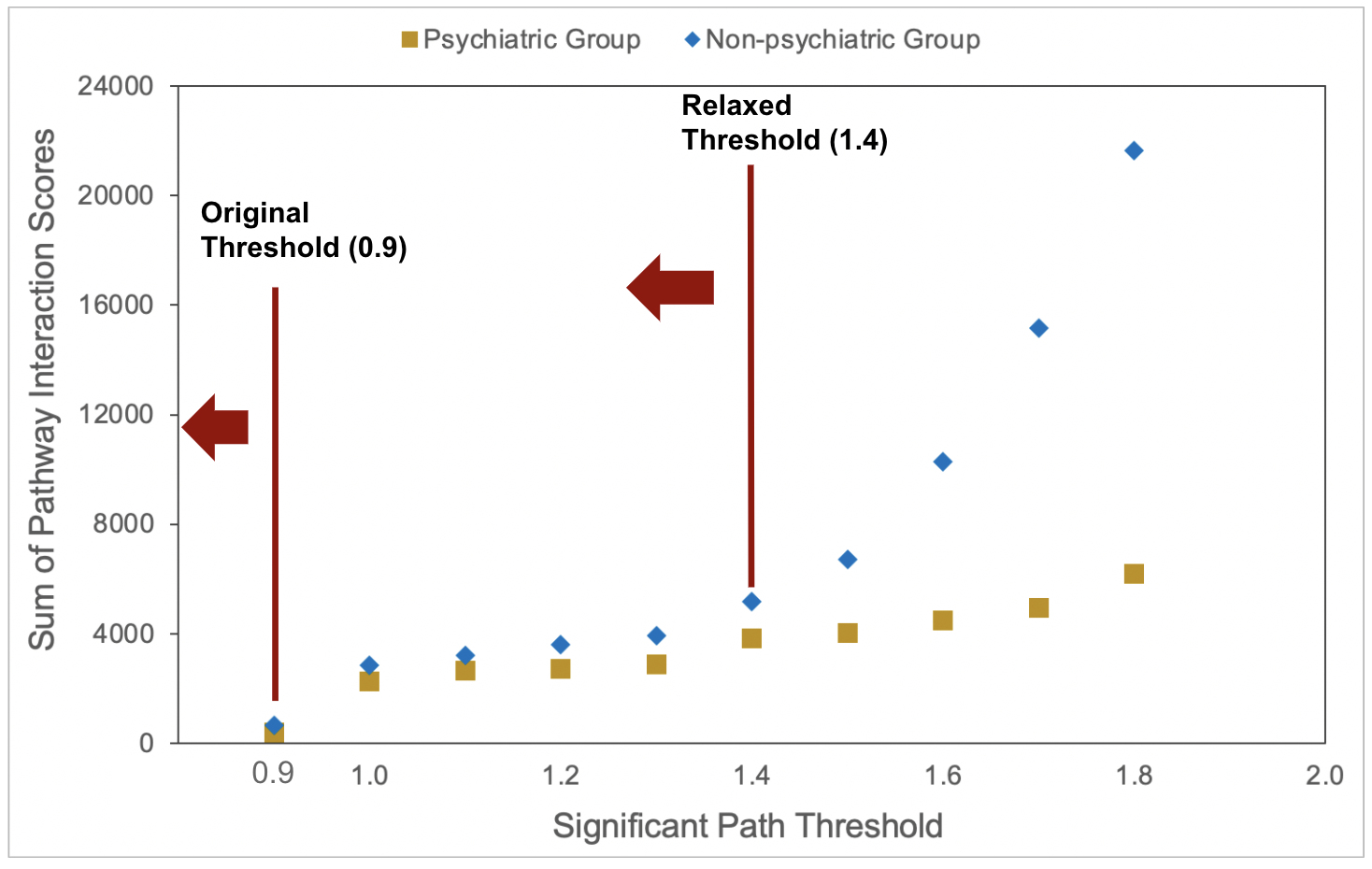

Supplement: Supplementary file 3 — Supplementary Material 3 [file 13023_2023_2953_MOESM3_ESM.docx]
